# Rapid and Efficient Optimization of Poly(1,2-Ethanediol Citrate) Synthesis Based on Magic Squares’ Various Methods

**DOI:** 10.3390/gels9010030

**Published:** 2022-12-30

**Authors:** Joanna Howis, Aleksandra Bandzerewicz, Agnieszka Gadomska-Gajadhur

**Affiliations:** Faculty of Chemistry, Warsaw University of Technology, Noakowskiego 3, 00-664 Warsaw, Poland

**Keywords:** citric acid, poly(1,2-ethanediol citrate), polycondensation, optimization, biomaterials

## Abstract

New biomaterials among aliphatic polyesters are in demand due to their potential applications in tissue engineering. There is a challenge not only to design scaffolds to regenerate defects in load-bearing tissues but also to ensure a proper blood supply to the reconstructed tissues. Poly-(1,2-ethanediol citrate) is one of the novel citrate-based polymers that could have the desired properties for cell scaffold fabrication and for enhancing cell adhesion. Both citric acid and 1,2-ethanediol are used in medicine and are fully resorbable by cells. This work aimed to synthesize poly(1,2-ethanediol citrate) in a catalyzed reaction with water removed by the Dean–Stark apparatus. The polyester structure was characterized by FTIR and NMR spectroscopy, and the HMBC experiment was performed to support the theory of successful polymer synthesis. The molecular weight was determined for the products obtained at 140 °C. The process was described via non-linear mathematical models. The influence of temperature and catalyst content on the degree of esterification and the conversion of acid groups in citric acid is described. The optimal process parameters are determined at 140 °C and 3.6% of p-toluenesulfonic acid content. The presented results are the starting point for scaffold design and scaling-up the process.

## 1. Introduction

In bioengineering, selecting suitable materials for a specific application is the key to success. Aliphatic polyesters possess desirable properties for biomedical applications, such as biocompatibility, biodegradability, and non-toxic degradation products [1]. Different polyesters have also received attention due to the reversibility of the esterification reaction in which they are obtained [2].

Nowadays, polylactide (PLA) is considered to be a valuable biodegradable polyester [3]. Its potential applications include bone grafting [4,5], ureteral stents [6] and controlled delivery carriers [7,8]. Nevertheless, PLA in biomedical applications may have limitations, such as poor hydrophilicity and slow degradation kinetics [9]. A relatively new and attractive polymer material is poly(glycerol sebacate) (PGS) which can interact with living cells [10]. The synthesis conditions determine its unique mechanical properties, opening up a wide range of possibilities for potential uses in soft tissues reconstruction [11,12]. PGS could be used to rebuild the myocardium and blood vessels [13,14] and eardrum [15] or act as a surgical sealer [16].

Despite numerous pieces of research showing the potential applications of PGS and PLA scaffolds for the regeneration of defects in load-bearing tissues, such as bones or cartilage, there is still concern about improving cell adhesion and growth. Modifying the process conditions, i.e., the ratio of glycerol and sebacic acid in PGS synthesis makes the material more hydrophilic; hence, the polymer shows better cell adhesion [17]. However, the change in substrates ratio also affects other properties such as the degradation time and mechanical strength, which is not desirable in scaffold fabrication [18,19].

The enormous challenge of providing adequate vascularization to support the viability, growth, and functionality of tissue substitutes that require a blood vessel supply is still a significant concern in tissue engineering. At this time, with available techniques, there is a possibility of regenerating tissues with low metabolic rates, such as cartilage, bone, and skin. Nevertheless, vascular networks are critical for efficiently distributing oxygen and nutrients in all tissues [20,21].

In response to today’s challenges in regenerative medicine, Yang et al. reported a new class of biomaterials [22]. It consists of copolymers of citric acid with aliphatic diols (C4-C12). The control of the mechanical properties of poly(diol citrates) is possible through the selection of diols and the conditions of post-polymerization processes. The citrate-based materials cover a wide range of mechanical properties, degradation profiles, and surface energies, which are crucial in controlling the biological response to an implanted material. The increased chain length in aliphatic dihydroxyl alcohol increases the polymer’s elasticity and decreases its density. Conversely, shortening the carbon chain, and thus, using more hydrophilic diol, results in stiffer materials and an increased degradation rate [23]. Citric acid is approved by the FDA and used, among others, in the food industry [24]. Poly(diol citrates), in preliminary in vitro and in vivo tests, are confirmed as ‘‘cell- and tissue-friendly’’ materials compatible with vascular cells and subcutaneous tissue [25].

Both citric acid and diols are multifunctional compounds, and this is favorable for further post-processing processes. Pendant functional groups are sacrificed for thermal polymer chain cross-linking [23]. The cross-linking density influences the molecular weight of the polymer and enables viscous liquids, sticky waxes, or even solids to be obtained. In the context of increasing demands in the area of tissue engineering, the multifunctionality of citric acid is also relevant given the design of biomaterials with unique features, i.e., antioxidant [26], antimicrobial, adhesive [27], and fluorescent properties [28].

The success of using polyesters obtained from citric acid and diols also prompted the use of trihydroxy alcohols. The ester groups in aliphatic polyesters with embedded glycerol units are separated with short carbon chains. Hence, the degradation rate is tailored to applications in bioengineering [29]. The difference in the reactivity of carboxyl acid groups in citric acid and hydroxyl groups in glycerol results in poly(glycerol citrate) with varying degrees of branching [30]. There is no need to use neither potentially cytotoxic solvents nor catalysts in the synthesis of poly(glycerol citrate) [31]. Poly(glycerol citrate)-polylactide nonwovens produced by electrospinning have properties similar to collagen fibers [32].

An alternative way of synthesizing citrate-based polyesters is transesterification. Nevertheless, in this case, there is no literature concerning this research area and the problem of synthesis optimization.

Although several biodegradable elastomers have been developed recently, most require time-consuming and complex synthesis that limits the possibility of scaling up the process [33]. Using the statistical design of experiment (DoE) early on in process development shortens the number and the cost of experiments and detects how interactions between factors can affect product yield and quality [34].

Currently, no literature is focusing on poly(1,2-ethanediol citrate). Nevertheless, due to the similarity in the chain length of glycerol and 1,2-ethanediol, the performance properties of their polyesters with citric acid should be comparable. Both citric acid and 1,2-ethanediol are easily accessible and are used in medicine; hence, they exhibit a high potential as substrates for biomedical materials. Given the lower functionality of the diol molecule (two hydroxyl groups instead of three), such a process should be more manageable. The lower branching of the resulting product should make it easier to predict the gel point of the reaction mixture. Thus, scaling up the process will be safer. Further research into using the obtained polyester to produce cell scaffolds will assess how the type of polyols used affects the cytotoxicity of the material.

The increasing interest in citrate-based polymers was the reason for investigating the catalyzed polycondensation of citric acid and 1,2-ethanediol. The work aimed to determine the optimal conditions in the synthesis of poly(1,2-ethanediol citrate) pre-polymer for further post-polymerization processes. We decided to maximize the degree of esterification to obtain polyesters with a high molecular mass. The influence of temperature and catalyst content on the properties of the obtained polymers was checked. The products were identified with spectral analysis and described with mathematical models.

## 2. Results and Discussion

### 2.1. Spectral Analysis of Synthesis Products

**Scheme 1:**
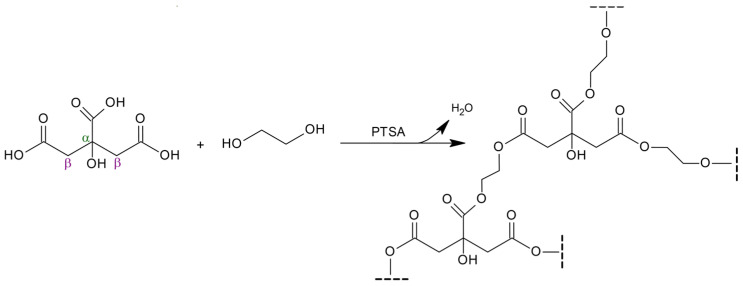
Synthesis of poly(1,2-ethanediol citrate).

Fourier transform infrared spectroscopy (FTIR) and ^13^C-NOE nuclear magnetic resonance (^13^C-NOE NMR) directly characterized the structures of the synthesized polymers. This paper adopted the following terminology to simplify the record and avoid misunderstandings. For the structure of citric acid and its derivatives, the carbonyl group bound to the α-carbon is referred to as α-C(O)O-H/R, and the carbonyl group bound to the β-carbon is referred to as β-C(O)O-H/R (Scheme 1).

**Figure 1:**
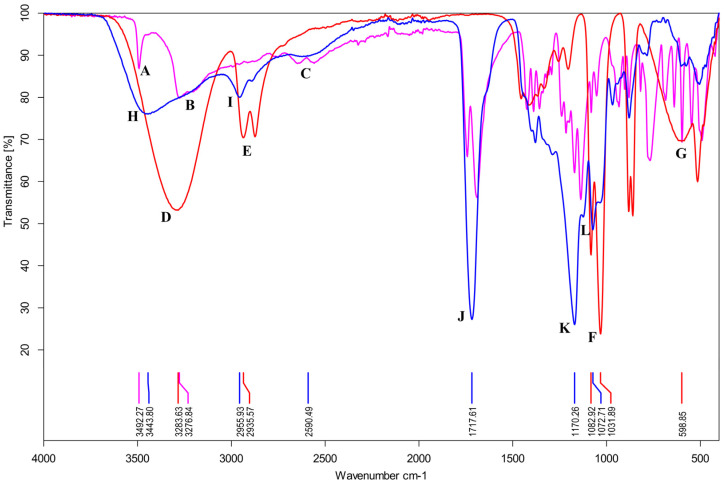
FTIR spectra of 1,2-ethanediol (red), citric acid (pink), and poly(1,2-ethanediol citrate) (blue).

Analysis of the characteristic bands on the FTIR spectrum shows that polyesters were obtained in the process. The formation of compounds with the polyesters’ structure was confirmed by comparison of the characteristic bands for poly(1,2-ethanediol citrate) with spectra of the substrates. (Figure 1). The obtained spectrum shows:

- A narrow band around 3500 cm^−1^ (A) corresponding to the vibrations of stretching O-H bonds not participating in the formation of the hydrogen bond in citric acid;
- A wide band in the range 3250–2600 cm^−1^ (B-C) derived from the hydroxyl groups in citric acid correlated with the hydrogen bond; an extensive band resulting from overlapping a band from a single O-H bond, hydroxyl groups in carboxyl moiety, and also a band corresponding to the vibrations of stretching C-H bonds of aliphatic groups; and the C band in the product spectrum indicates the incomplete conversion of citric acid;
- Characteristic broad band of high intensity in the range 3600–3100 cm^−1^ (D) of the O-H stretching vibrations in 1,2-ethanediol;
- An extensive band in the range 3650–3200 cm^−1^ (H) corresponds to the stretching vibrations of the hydroxyl groups of both 1,2-ethanediol (D) and citric acid (A, B), resulting from the incomplete conversion of the functional groups in poly(1,2-ethanediol citrate);
- A band of about 2950 cm^−1^ is derived from the vibrations of stretching C-H bonds of the aliphatic groups in 1,2-ethanediol (E) as well as in the reaction products, i.e., the polymer chain and substrate molecules (I);
- A band with a maximum at 1720 cm^−1^ (J) characteristic of the stretching vibrations of the carbonyl groups in acids and esters; the double band in citric acid indicates the inequality of the carboxyl groups or may result from the presence of hydrogen bonds and the formation of associations; in addition, a slight widening on the right side of the ester band can be noticed, which proves incomplete conversion of the acid;
- A strong band around 1050 cm^−1^ (F) of the C-O stretching vibrations in the C-C(O)-O group in 1,2-ethanediol; in the case of the ester, a shift of the C-O band to 1170 cm^−1^ (K) is observed;
- A broad band corresponding to the deformation vibrations of the hydroxyl groups related to hydrogen bonding in 1,2-ethanediol in the range 800–550 cm^−1^ (G);
- A band around 1080 cm^−1^ (L) derived from C-O stretching vibrations in the O-C-C group.

The absence or distortion of many of the bands on the poly(1,2-ethanediol citrate) spectrum present in the case of citric acid and 1,2-ethanediol indicates that the substrates have reacted and a polyester-structured compound has been formed. In particular, this is supported by the changes within the bands in the range 3500–3000 cm^−1^ of hydroxyl groups. This change is due to the formation of ester bonds. Additionally, the characteristic bands for carbonyl groups in the esters appear to be about 1700 cm^−1,^ and the band from the C-O stretching vibrations in the C-C(O)-O group is shifted towards lower ranges. The discussed relationships confirm the positive synthesis of a compound with a polyester structure.

**Figure 2:**
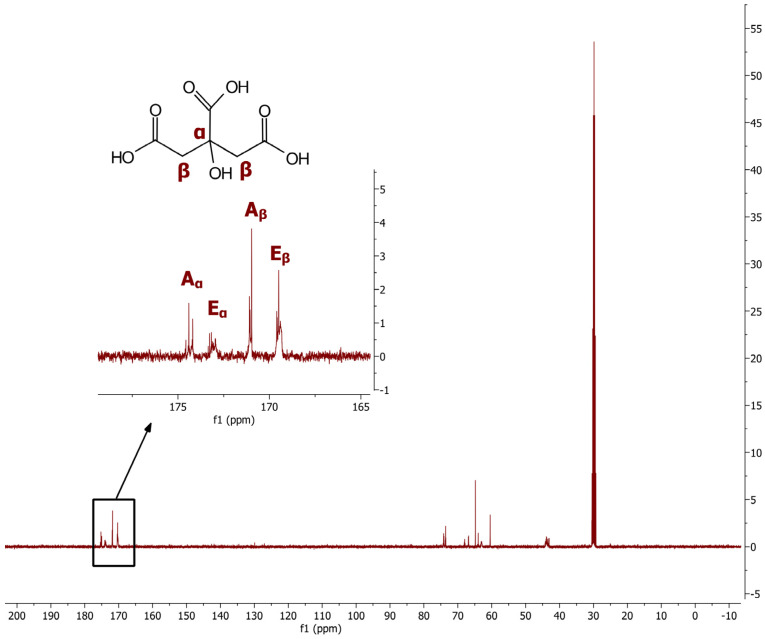
^13^C−NOE NMR spectrum of poly(1,2-ethanediol citrate).

The polyester formation may be affirmed in detail based on the characteristic range of carbonyl carbons in the ^13^C-NOE NMR. As shown in Figure 2, four-carbon multiples of different intensities were observed. The signal at δ174 ppm belongs to α-C(O)O-H, and the signal at δ171 ppm belongs to the β-C(O)O-H groups. Additionally, the proportion of stoichiometrically twice the excess of β-carbons relative to α-carbons may be observed in the signals’ intensities. The signals at δ173 ppm and δ169 ppm correspond respectively to the α-C(O)O-R and β-C(O)O-R groups. Gadomska-Gajadhur et al. confirmed with the ^1^H−^13^C carbonyl carbon atoms’ correlation spectrum that the signals of the carbonyl groups in esters are shifted towards lower ppm values relative to the carbonyl groups of acid [31]. The carbonyl carbons of carboxylic acid derivatives, e.g., esters, are strongly deshielded (165–180 ppm) due to the close presence of highly electronegative oxygen. Based on the differences in the values of the chemical shifts of the signals of the α-C(O)O-H and β-C(O)O-H carbon atoms for pure citric acid and synthesized product, complete conversion of citric acid molecules was confirmed. The signals from the carboxylic groups were derived from incompletely converted acid groups in the polymer chain.

**Figure 3:**
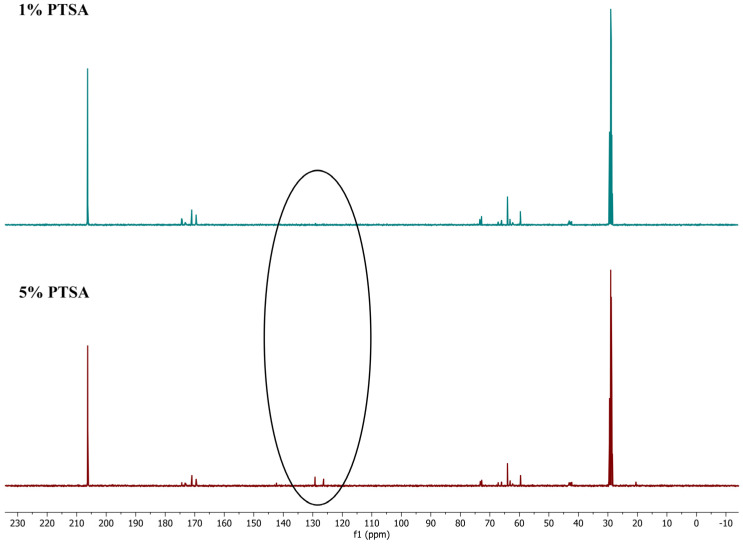
^3^C−NOE NMR spectra of selected poly(1,2-ethanediol) samples with different catalyst content.

Additionally, the characteristic peaks in the range δ125–δ130 ppm were noticed for some polyester samples synthesized with a catalyst content exceeding 1% of the citric acid mass content (Figure 3). The discussed relationship was also apparent in the slightly yellow color of these samples. In conclusion, the increase in the catalyst content apparently promotes dehydration.

**Figure 4:**
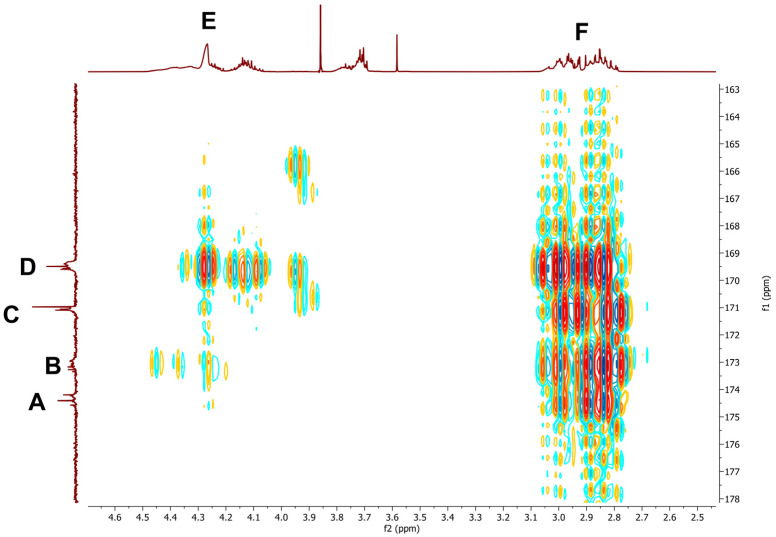
HMBC spectrum of the poly(1,2-ethanediol citrate)—a ^1^H–^13^C carbonyl carbon atoms’ correlation fragment.

The heteronuclear multiple bond coherence (HMBC) experiment (Figure 4), which gives correlations between ^1^H and ^13^C nuclei separated by several bonds, shows the interconnection of the protons of the methylene groups in acid-derived ester fragments (signal F) and the carbon atoms of the α-C(O)O-H (signal A), and α-C(O)O-R (signal B), and β-C(O)O-H (signal C), and β-C(O)O-R (signal D). Moreover, the signal of the protons derived from the ethanediol fragments in the ester is shifted at δ4.0–δ4.4 ppm (signal E) due to the coupling with the α-C(O)O-R and β-C(O)O-R groups. In the ethylene glycol, the signal is observed at δ3.7 ppm. These findings support the theory that poly(1,2-ethanediol citrate) was synthesized with success.

### 2.2. Optimization of the Synthesis Conditions

**Figure 5:**
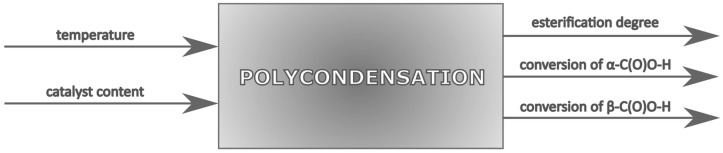
“Black box” for the optimized process.

This paper used an optimization technique based on the magic square plan to investigate the effect of optimized parameters on pre-polymer molecular weight. The synthesis of poly(1,2-ethanediol citrate) was optimized with the design of experimental (DOE) methods. Conducting a limited number of experiments enabled a reliable synthesis model to be obtained. The mathematical models describing the change in the ester degree (ED) and conversion of α-C(O)O-H (X_α_^NMR^) and β-C(O)O-H (X_β_^NMR^) acid groups were used to find the optimal conditions for the synthesis process. The input variables were the reaction temperature and the catalyst content, depending on the mass of the citric acid used in the polycondensation. The discussed process is presented as a “black box” in Figure 5.

On the basis of preliminary research, the process variables and their values were determined. The most favorable is polymerization while maintaining an equimolar ratio of the moieties. However, the negligible availability of the hydroxyl group (spatial hindrances) in citric acid was not considered when determining the molar ratio. Due to the process’ limitations, upper and lower limits were established for the other synthesis parameters. The thermal decomposition of citric acid determines the upper limit at 175 °C. As for the temperature, the reaction should not have proceeded under 100 °C because water is removed from the process. In the most extreme case, the polycondensation could be solvent-free, complying with the principles of green chemistry.

On the other hand, the more catalyst, the greater risk of side reactions, including dehydration. In this paper, the following input variables were adopted for optimization: citric acid/ethylene glycol 2:3 (molar ratio of COOH/OH 1:1), temperature: 120–140 °C, and the catalyst content 0–5%. The reaction time was established as 60 min, including 10 min of reaching the set temperature.

The performance properties of the obtained products varied depending on the reaction parameters. There were visible differences in the viscosity and the color of the samples, from colorless semi-liquids to light yellow resins. Brief descriptive characteristics of the materials are outlined in Table 1.

The GPC analysis was initially performed for products obtained at 140 °C. According to the authors’ knowledge, it was assumed that in the experiment area, the polymer molecular weight would be the highest for the maximum temperature. The results of the polyester molecular weight are presented in Table 2.

Surprisingly low molecular weight results may arise from obtaining poly(1,2-ethanediol citrate) as oligomers. The synthesis ought to result in a polymer characterized by a high molecular weight. Despite that assumption, the synthesis of oligomers is not considered a significant drawback due to the further cross-linking step.

Another explanation of this phenomenon is the imprecision of the GPC method and linear PEG standards, which are unsuitable for testing branched structures such as poly(1,2-ethanediol citrate).

The discussed problem was also presented in the studies on synthesizing glycerol and citric acid [31]. Taking into consideration the questionable outcome of the polymer molecular weight and the irrelevance of these results for further work, it was decided for them not to be included in the optimization. The mathematical model for molecular weight is not presented in this paper.

**Figure 6:**
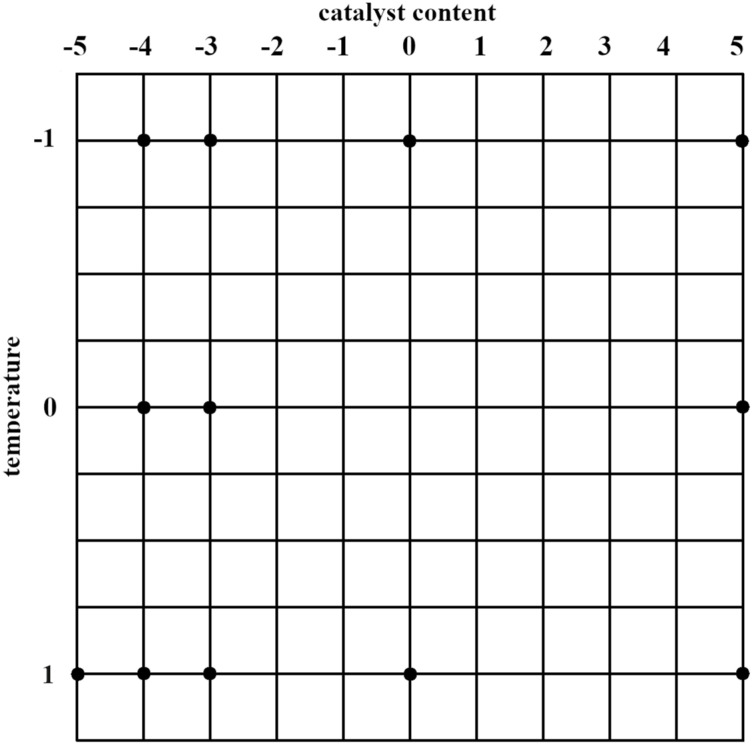
The selection of input variables by magic squares various methods.

The mathematical models describing temperature dependence and the catalyst content, built on the experimental data, were determined using StatSoft Statistica. In all instances, the quadratic equations with the factor of synergism of variables were obtained. The experiments were planned on a square plan to simplify the statistical analysis and eliminate the row and column variation of the experimental results (Figure 6). The design of experiments included nine coded experiments on the sides of the square and one following corresponding to a non-catalyzed reaction. To dispel doubts about the fitting of the model in the original square plan, two additional experiments were performed in the central part of the plan (Table 3—N^o^ 8–9). The input variables were presented in a codded manner to correctly determine their impact on the process (Table 4).

The following equations were obtained:

(1) $$ED\;\left[\%\right]=61.39+4.05\left(T\right)+0.89\left({\%}_{cat}\right)+0.59{\left(T\right)}^{2}-0.23{\left({\%}_{cat}\right)}^{2}+0.08\left(T\right)\left({\%}_{cat}\right)$$

(2) $$X_{\alpha }^{NMR}\;\left[\%\right]=58.37+7.68\left(T\right)+3.00\left({\%}_{cat}\right)+0.29{\left(T\right)}^{2}-0.63{\left({\%}_{cat}\right)}^{2}+0.56\left(T\right)\left({\%}_{cat}\right)$$

(3) $$X_{\beta }^{NMR}\;\left[\%\right]=63.71+3,44\left(T\right)+0.16\left({\%}_{cat}\right)+1.37{\left(T\right)}^{2}-0.24{\left({\%}_{cat}\right)}^{2}+0.50\left(T\right)\left({\%}_{cat}\right)$$

**Figure 7:**
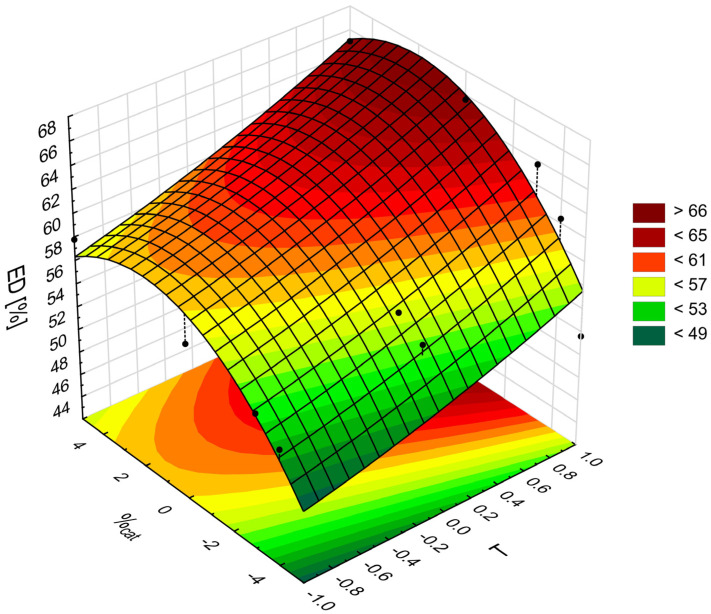
The ED variability depending on the reaction temperature (T) and the catalyst content (%_cat_).

**Figure 8:**
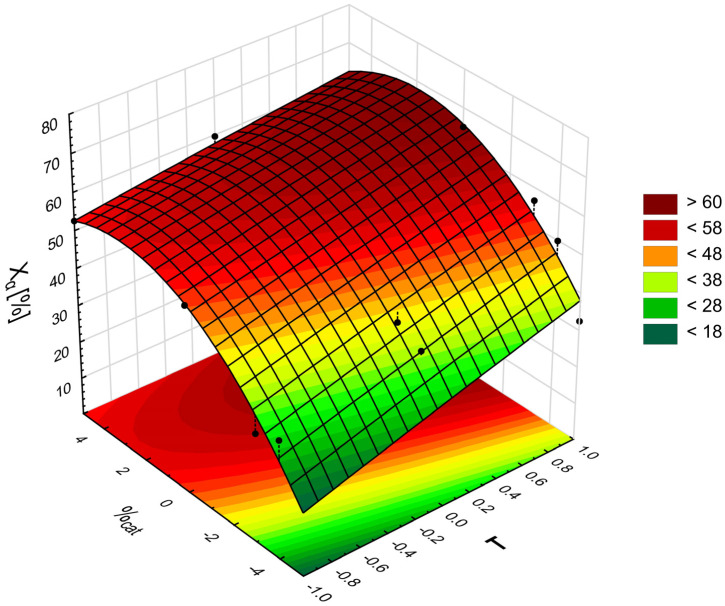
The X_α_^NMR^ variability depending on the reaction temperature (T) and the catalyst content (%_cat_).

**Figure 9:**
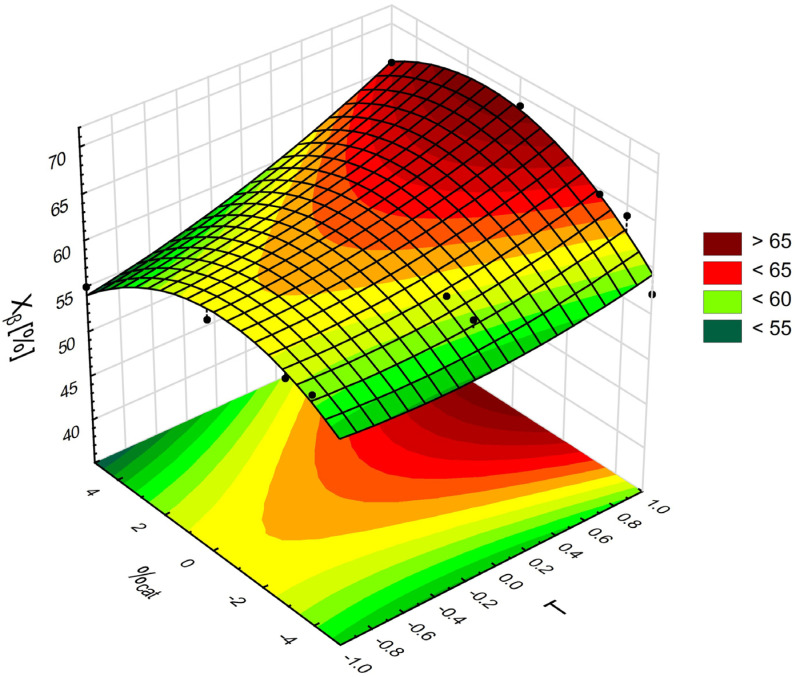
The of X_β_^NMR^ variability depending on the reaction temperature (T) and the catalyst content (%_cat_).

The variability of the process depending on the temperature and catalyst content is visualized as response surfaces (Figure 7, Figure 8 and Figure 9).

The residual coefficients of variation and determination determine the quality of the model fit to the experimental values (Table 5). As the residual variation tends to zero, the best fit is in the conversion model of the β-C(O)O-H acid group (X_β_^NMR^). The high value of the coefficient of determination also confirms it. The compliance of all of the presented mathematical models is sufficient to recognize their usefulness in scaling-up the process.

Both the presented equations and the response surfaces are designated for standardized (coded) input variables and require translation into the values of normed variables in the experimental area. Operating normalized dimensionless variables allows us to directly evaluate the effect of given parameters based on appropriate coefficients. For all equations, the most influential is the constant term, which suggests the occurrence of other important variables influencing the process, not included in this optimization.

The presented response surfaces allow several conclusions to be drawn about the course of the process. The temperature has a much more significant effect on the conversion of α-C(O)O-H groups than β-C(O)O-H. This may indicate that as the process temperature increases, the selectivity of the attack of the hydroxyl group of the diol on the carboxyl group decreases. The steric hindrance then becomes less significant. It is also associated with a greater risk of gelling of the reaction mixture, for which esterification of the α-C(O)O-H groups is mainly responsible. However, it should be noted that the positive temperature effect for α-C(O)O-H groups occurs when the coded value of this variable is above zero. Otherwise, the linear and quadratic effects will cancel each other out. The case is similar for the conversion of β-C(O)O-H. However, the higher value of the intercept of the equation and the lower values of the coefficients at both temperature factors results in a more significant contribution of β-C(O)O-H groups to the esterification process than α-C(O)O-H groups in the temperature below 130 °C. Again, this is most likely due to the effect of steric hindrance and the lower reaction rate. The maximum value can be obtained by establishing the highest temperature within the explored area according to all the response surfaces. These conclusions are consistent with the preliminary studies. Increasing the temperature for a given reaction time increases the conversion ratio.

Nevertheless, introducing the catalyst into the process may obtain the same product using a lower temperature. The product obtained in a non-catalyzed reaction at 140 °C should have the same performance properties as that obtained at 120 °C with a 0.5% catalyst. On the other hand, it should be pointed out that the increase in catalyst content affects the color of the polymer.

An increase in the catalyst content above around 3.5% results in a progressive decrease in ED. The most probable cause of this phenomenon is a catalyst promoting acidic dehydration. Due to the process’s complexity, the conversion ratio increase is observed only in the specified range of variability of input variables.

Moreover, by analyzing Table 2 and the mathematical models of α-C(O)O-H (X_α_^NMR^) and β-C(O)O-H (X_β_^NMR^) acid groups’ conversion, it could be assumed that the higher temperature and the higher catalyst content favor the conversion of α-C(O)O-H groups. In addition, considering the relatively high conversion of β-C(O)O-H groups, it appears that a branched structure of the polymer is obtained under these conditions. The conversion of β-C(O)O-H groups is comparable to the catalyzed reactions for the catalyst-free synthesis. Still, the conversion of α-C(O)O-H groups is dramatically reduced. These conclusions indicate obtaining the linear structure of the polymer.

This work aimed to find the optimal parameters of the process. The main optimization criterion was the ED’s maximization, not the total conversion of X^NMR^. Although both parameters refer to the same phenomenon, the error in NMR analysis is more significant.

The following optimal process parameters were designated: 140 °C and 3.6% catalyst content. The synthesis performed under the optimal conditions confirmed the model’s good fit—the values predicted with the mathematical model do not differ significantly from real ones (Table 2—N^o^ 14). It is worth noting that the obtained polymer was slightly yellow.

In scaling-up the process, it should be taken into account that reducing the temperature and catalyst content compared to the optimal determined values might be favorable for obtaining the linear structure of the polymer. An increase in the degree of branching of the polymer above the critical value may result in the gelling effect, which is particularly undesirable in the technological process. More detailed research in this area should be completed. Nevertheless, the presented mathematical models can be considered helpful in scaling-up the synthesis of poly(1,2-ethanediol citrate) and will be further investigated for biomedical applications.

## 3. Materials and Methods

Commercially available ethanediol (Fisher Chemical, >99%), anhydrous citric acid (Acros Organics, ≥99.5%), and p-toluenesulfonic acid monohydrate—PTSA (Sigma Aldrich, ≥98.5%) were used without prior preparation.

### 3.1. Fourier Transform Infrared Spectroscopy (FTIR)

A Bruker FTIR ALPHA II spectrometer was used to obtain Fourier transform infrared (FTIR) spectra at room temperature. The method performed thirty-two scans in the range of 400–4000 cm^−1^ each time.

### 3.2. Nuclear Magnetic Resonance (NMR)

An Agilent 400 MHz NMR spectrometer was used to record the spectra. The samples were prepared by dissolving about 150 mg of poly(1,2-ethanediol citrate) in 1 mL of acetone for 24 h. MestReNova NMR software was used for data processing.

The following formulas were applied for calculations of the degree of conversion of the carboxyl groups of citric acid:

(4) $$X_{\alpha }^{NMR}=\frac{\int E_{\alpha }}{\int E_{\alpha }+\int A_{\alpha }}\cdot 100\%$$

(5) $$\;X_{\beta }^{NMR}=\frac{\int E_{\beta }}{\int E_{\beta }+\int A_{\beta }}\cdot 100\%$$

(6) $$X^{NMR}=\frac{\int E_{\alpha }+\int E_{\beta }}{\int E_{\alpha }+\int A_{\alpha }+\int E_{\beta }+\int A_{\beta }}\cdot 100\%$$

where:

$X_{\alpha }^{NMR}$—the conversion of α-C(O)O-H acid groups;
$X_{\beta }^{NMR}$—the conversion of β-C(O)O-H acid groups;
$X^{NMR}$—the total conversion of the acid groups;
$\int E_{\alpha },\int E_{\beta }$—the value of the integral of the signal from the ester group α-C(O)O-R or β-C(O)O-R;
$\int A_{\alpha },\int A_{\beta }$—the value of the integral of the signal from the acid group α-C(O)O-H or β-C(O)O-H.

### 3.3. Gel Permeation Chromatography (GPC)

The molecular weight of poly(1,2-ethanediol citrate) was determined with the HPLC easy mate 3000 apparatus (pre-column and two Tosoh Bioscience columns cat. 17368 and 17355) equipped with a Shodex RI-101 refractive index detector with a flow ratio of 0.8 mL/min at 30 °C. About 60 mg of each sample was weighed into a 10 mL heart flask, and 5 mL of THF was added. The samples were dissolved for 24 h at 30 °C. The solutions were filtered using 45 μm syringe filters. Curve calibration was performed using PEG standards (Easy Vials, Agilent).

### 3.4. Acid Number

About 1–1,5 g of the sample was weighed and dissolved in 25 mL of methanol. Four drops of indicator—thymol blue—for each trial were added. The samples were titrated with 1 M NaOH_(aq)_ until the change in color from yellow to blue was observed. The result is a mean of two/three determinations for each sample of poly(1,2-ethanediol citrate). A blank test was performed under the same conditions. The acid number (AN) was calculated by using the following equation:

(7) ANmgKOHgsample=V−Vo⋅MNaOH⋅56.1m

where:

V—the volume of 1 M NaOH aqueous solution consumed in the actual test;
$V_{o}$—the volume of 1 M NaOH aqueous solution consumed in the blank test;
$M_{NaOH}$—the titer of solution used for titration (1 M);
m—the sample weight.

### 3.5. Ester Number

About 1–1,5 g of the sample was weighed and dissolved in a solution of 15 mL of methanol and 20 mL of 1 M aqueous NaOH. Then, the solutions were refluxed for 1 h at a temperature of around 150 °C. The solutions were left to cool, and then four drops of phenolphthalein indicator were added for each trial. The samples were titrated with 1 M HCl_(aq)_ until discoloration of the pink solution. A blank test was performed under the same conditions. The result is a mean of two/three determinations for each sample of poly(1,2-ethanediol citrate). The ester number (EN) was calculated by using the following equation:

(8) ENmgKOHgsample=Vo−V⋅MHCl⋅56.1m−AN

where:

V—the volume of 1 M HCl aqueous solution consumed in the actual test;
$V_{o}$—the volume of 1 M HCl aqueous solution consumed in the blank test;
$M_{HCl}$—the titer of solution used for titration (1 M);
m—the sample weight.

The ester degree (ED) was calculated by using the following equation:

(9) $$ED=\frac{EN}{EN+AN}\cdot 100\%$$

where:

EN—ester number;
AN—acid number.

### 3.6. Synthesis Procedure

Poly(1,2-ethanediol citrate) was synthesized in the MultiMax Mettler Toledo reactor systems, in Hastelloy reactors (50 mL) equipped with a mechanical stirrer, a temperature sensor, and Dean–Stark apparatus. The anhydrous citric acid (28.83 g; 0.15 mol), ethanediol (13.97 g; 0.225 mol), and p-toluenesulfonic acid monohydrate (0–1.4415 g; 0–0.0076 mol) were weighed into the reactor. Initially, the reaction mixture was heated to the set point temperature (120–140 °C) for 10 min. Once the set temperature had been reached, the set point temperature was held constant for 50 min, and a Dean–Stark apparatus was used for water removal.

StatSoft Statistica software was used for graphics and calculations preparation.

## 4. Conclusions

The polycondensation of citric acid with 1,2-ethanediol was investigated and described with mathematical models, which allow the prediction of product properties depending on the temperature and catalyst content. Polyester formation was confirmed with detailed spectral analysis, including FTIR and NMR correlations.

The matrix equations determined the optimal process parameters at 140 °C and 3.6% of p-toluenesulfonic acid content. The high temperature favored the maximization of the esterification degree. Nevertheless, introducing a catalyst into the process lowers the temperature needed for obtaining the product with comparable performance properties. Although non-catalyzed reactions are safer for biomedical applications, adding the catalyst reduces energy consumption, and hence, the economy of the process is more favorable.

Regenerative medicine requires using multifunctional materials covering a wide range of mechanical properties. Poly(1,2-ethanediol citrate) meets these requirements. What is more, considering its potential for functionalization, scientist should consider poly(1,2-ethanediol citrate) as a potential drug delivery carrier of interest. Finally, considering the growing interest in aliphatic polyesters for scaffold design, polyesters of citric acid will undoubtedly attract attention.

## Figures and Tables

**Table 1:** The performance features of the reaction products at room temperature.

		Temperature [°C]		
		120	130	140
**Catalyst content [%cat]**	**0**	-	-	Colorless, viscous semi-liquid
	**0.5**	Colorless, viscous semi-liquid	Colorless, viscous semi-liquid	Colorless, sticky, hardening into resin
	**1**			Colorless, sticky, fiber-forming resin
	**2.5**	Light yellow, viscous semi-liquid	-	
	**5**		Light yellow, viscous semi-liquid	

**Table 2:** The molecular weight of poly(1,2-ethanediol citrate) pre-polymers was obtained at 140 °C.

N^o^	Catalyst Content [%_cat_]	*M*_n_^GPC^ [Da]	*M*_w_^GPC^ [Da]	PDI
1.	0	392	413	1.05
2.	0.5	613	665	1.09
3.	1	478	562	1.17
4.	5	570	699	1.22

**Table 3:** The comparison of the optimization results (exp.) with calculated values (cal.) derived from the analysis of the synthesis products.

N^o^	T [°C]	Catalyst Content [%_cat_]	ED [%]		X_α_^NMR^ [%]		X_β_^NMR^ [%]	
			Exp.	Cal.	Exp.	Cal.	Exp.	Cal.
1.	0	0	52	55	32	33	57	59
2.	120	0.5	52	51	32	31	60	59
3.	130		55	54	37	36	60	59
4.	140		60	58	50	42	64	62
5.	120	1	54	53	29	38	60	61
6.	130		56	57	41	44	61	61
7.	140		64	61	57	50	64	64
8.	120	2.5	55	58	50	51	60	62
9.	140		65	66	65	66	69	69
10.	120	5	65	67	68	71	68	69
11.	130		58	56	53	47	55	54
12.	140		59	60	60	58	58	59
13.	140	3.6	65	67	68	71	68	69

**Table 4:** Values of coded input variables.

Input Variable	Lower Limit Value	(−4.00)	(−3.00)	(−1.00)	0	(+1.00)	(+5.00)	Upper Limit Value
Temperature [°C]	100	-	-	120	130	140	-	170
Catalyst content [%_cat_]	0	0.5	1	-	2.5	-	5	-

**Table 5:** The statistical analysis of the presented mathematical models.

Coefficient	Equation		
	ED	X_α_^NMR^	X_β_^NMR^
Residual variation	0.0328	0.1056	0.0178
Determination (R^2^)	0.8690	0.8628	0.9378

## Data Availability

Not applicable.
